# MRP4 regulates ENaC-dependent CREB/COX-2/PGE_2_ signaling during embryo implantation

**DOI:** 10.18632/oncotarget.19676

**Published:** 2017-07-28

**Authors:** Jun-Jiang Chen, Yan Wang, Xiaojing Meng, Ye Chun Ruan, Fei Zou, Hsiao Chang Chan

**Affiliations:** ^1^ Department of Occupational Health and Occupational Medicine, Guangdong Provincial Key Laboratory of Tropical Disease Research, School of Public Health, Southern Medical University, Guangzhou, China; ^2^ Epithelial Cell Biology Research Centre, School of Biomedical Sciences, Faculty of Medicine, The Chinese University of Hong Kong, Hong Kong, China; ^3^ Sichuan University – The Chinese University of Hong Kong Joint Laboratory for Reproductive Medicine, West China Second University Hospital, Sichuan University, Chengdu, China; ^4^ Shenzhen Research Institute, The Chinese University of Hong Kong, Hong Kong, China; ^5^ Interdisciplinary Division of Biomedical Engineering, Faculty of Engineering, The Hong Kong Polytechnic University, Hong Kong, China

**Keywords:** MRP4, embryo implantation, CREB, COX-2, PGE_2_

## Abstract

Multi-drug resistance protein 4 (MRP4), a potential chemotherapeutic target as well as a transporter for endogenous signaling molecules (e.g. prostaglandins), is known to be expressed in the endometrium, although its possible role(s) in the physiology of the endometrium remains unknown. Here, we show that MRP4 is upregulated at implantation window and localized to the basolateral membrane of the endometrial epithelium, the interface between the epithelium and stroma in mice. In human endometrial epithelial cells, MRP4 expression is upregulated by ENaC activation and the inhibition of MRP4 blocks ENaC-dependent PGE_2_ release as well as phosphorylation of CREB. Intrauterine injection of MRP4 inhibitor in mice prior to implantation significantly downregulated implantation markers COX-2, Claudin4 and Lif, and reduced implantation rate. These results in together have revealed a previously undefined role of MRP4 in mediating ENaC-dependent CREB/COX-2/PGE_2_ signaling essential to embryo implantation with implication in cancer progression as well.

## INTRODUCTION

Embryo implantation is a prerequisite for a successful pregnancy in mammals [1, 2]. Implantation failure accounts for the majority of pregnancy losses in humans [3] and aberrant implantation is believed to result in severe outcomes at later pregnancy stages including placental insufficiency, preeclampsia and preterm labour [1]. However, due to its complexity and technical limitations especially in humans, embryo implantation remains a poorly understood process.

Prostaglandins (PGs) are a group of bioactive lipid compounds long known to be required for embryo implantation. Deletion of enzymes for PGs synthesis including phospholipase A2 and cyclooxygenase-2 (COX-2) results in implantation failure in mice [4, 5]. In humans, exposure to non-steroid anti-inflammatory drugs that block PGs synthesis during pregnancy significantly increases the risk of miscarriage [6]. Also, impaired endometrial PGs synthesis was observed in women with repeatedly implantation failure in IVF trials [7]. Prostaglandin E2 (PGE_2_), mainly produced by COX-2-driven synthesis, is considered one of the most important PGs to initiate decidualization [5, 8], an endometrial stromal differentiation process required for embryo implantation [9]. We have previously demonstrated that the epithelial sodium channel (ENaC) in the endometrial epithelial cells can be activated by embryo-derived protease, which subsequently triggers a sequence of events in endometrial epithelial cells, including Ca^2+^ increase, phosphorylation of CREB (Ca^2+^/cAMP responsive element binding protein), downregulation of miR101 and miR199a, upregulation of COX-2 and eventually PGE_2_ production and release to the stroma, leading to decidualization and embryo implantation [10, 11]. However, it should be noted that the permeability of plasma membrane to PGE_2_ is low due to its negative charges [12, 13] and how PGE_2_ is released from the endometrial epithelial cells for induction of stromal decidualization required for embryo implantation remains unclear.

Multi-drug resistance protein 4 (MRP4), a member of the ATP-binding cassette (ABC) transporter family, is known to mediate the efflux of both exogenous drugs and endogenous molecules including PGs [14–17]. It has been reported that upregulation/overexpression of MRP4 in colorectal cancer tissues and cell lines is correlated to high extracellular level of PGE_2_, suggesting its capacity in transporting PGE_2_ [18]. Indeed, MRP4 has been reported to transport PGE_2_ in the bovine endometrium [19] and that MRP4 knockout mice showed reduced litter size [20]. Given the importance of ENaC-regulated COX-2/PGE_2_ signaling in embryo implantation and the PGE_2_-transporting capacity of MRP4, we hypothesized that MRP4 might be involved in mediating the ENaC-dependent signaling required for embryo implantation. We undertook the present study to test this possibility *in vitro* and *in vivo*.

## RESULTS

### Upregulation of MRP4 by ENaC activation during embryo implantation

**Figure 1:**
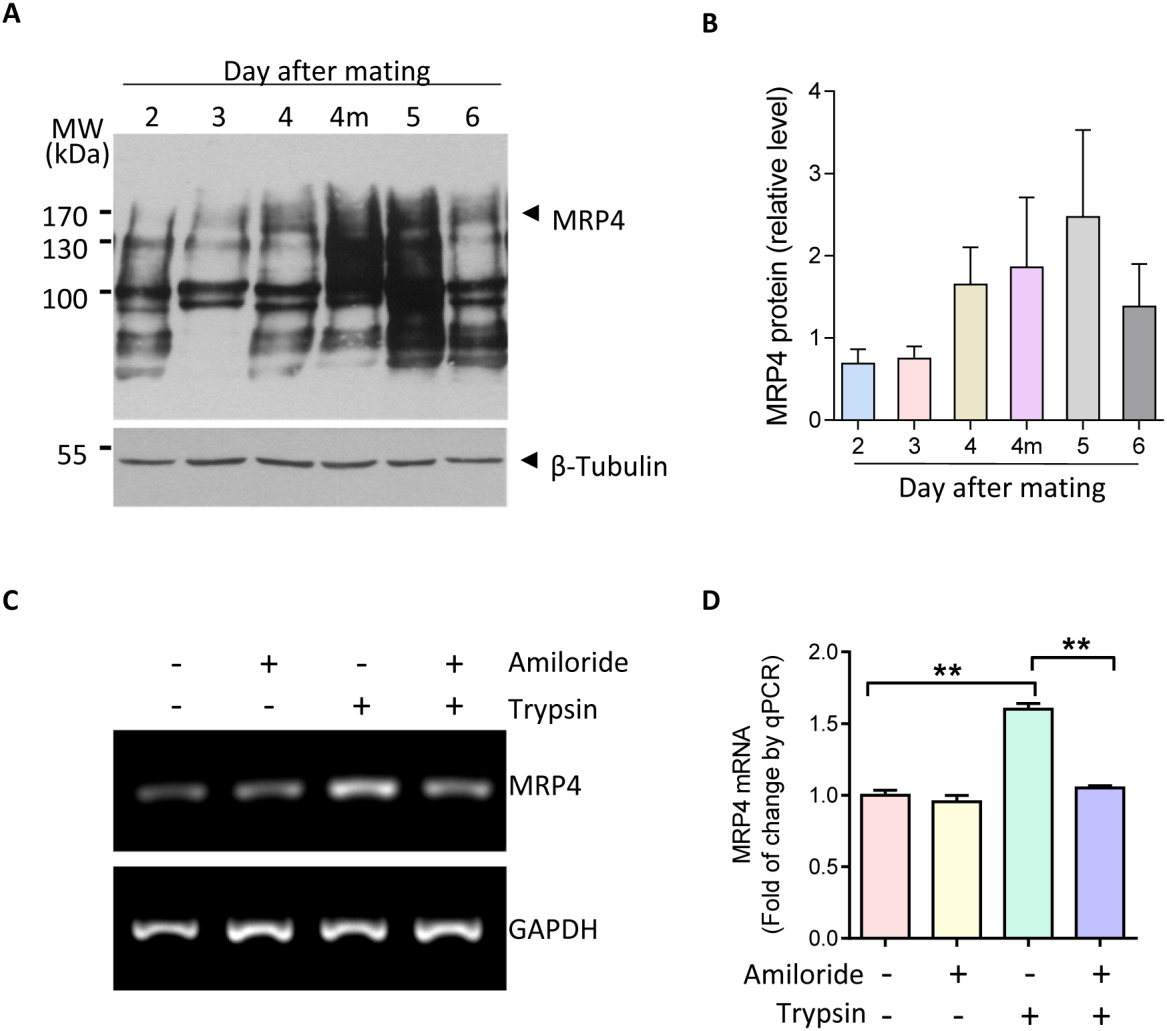
Upregulation MRP4 by ENaC activation during embryo implantation **(A-B)** Western blots **(A)** with quantification **(B)** for MRP4 in mouse uterus during the peri-implantation period from days 2-6 post mating (4m: midnight of day 4) (n = 3). **(C-D)** Conventional **(C)** and quantitative **(D)** PCR of MRP4 in human endometrial epithelial cells in response to trypsin (20 μg/ml, ENaC activator) in the absence or presence of amiloride (10 μM, ENaC blocker) (n = 3, ** P < 0.01).

We first examined the expression of MRP4 in mouse uterus at day 2 to 6 post mating and found that MRP4 was abundantly upregulated in midnight of day 4, when the implantation is initiated, and day 5, when decidualization begins in mice (Figure 1A&1B). This expression pattern of MRP4 is consistent with that previously reported for ENaC during embryo implantation [10], and thus we further examined whether the expression of MRP4 could possibly be altered by ENaC activation during embryo implantation. We used trypsin, an embryo-released protease known to activate ENaC, to treat human endometrial epithelial cells. As shown in Figure 1C&1D, activation of ENaC by trypsin (20 μg/ml, 15 mins) induced a significant increase of MRP4 at mRNA level in the cells, which could be abolished by pretreatment with amiloride (10 μM, 24 hours), a selective ENaC blocker, suggesting that MRP4 transcription could be activated by ENaC activation during embryo implantation.

### MRP4 mediates ENaC activation-triggered epithelial PGE_2_ release into stroma at embryo implantation

**Figure 2:**
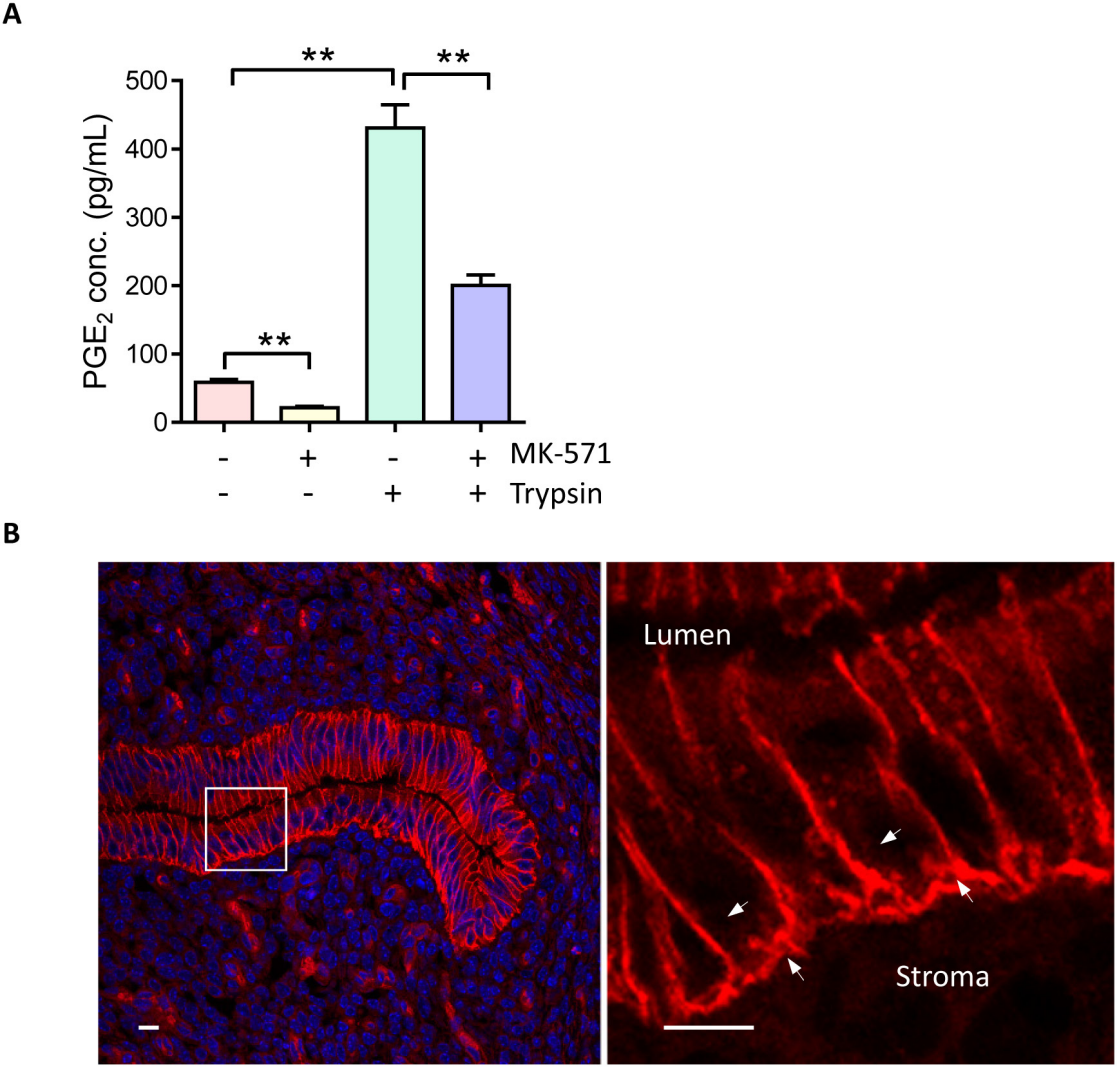
MPR4 mediates ENaC activation-triggered PGE_2_ release **(A)** ELISA detection of PGE_2_ levels in medium incubated with human endometrial epithelial cells with or without trypsin (20 μg/ml) and MK-571 (10 μM, MRP4 blocker) (n = 3, ** P < 0.01). **(B)** Immunofluorescence labeling (Red) for MRP4 in mouse uterus at day 4 post mating. Arrows: basolateral membrane of the endometrial epithelial cells. Lu: lumen. Nuclei were visualized by DAPI staining. Scale bar = 5 μm.

Given the reported capacity of MRP4 in transporting PGE_2_ uniquely among the ABC transporters [21], we next asked whether MRP4 could mediate the epithelial release of PGE_2_ triggered by ENaC activation during embryo implantation. We treated the human endometrial epithelial cells with MK-571 (10 μM, 24 hours), a selective inhibitor of MRP4 transporting function, and found that the concentration of PGE_2_ in the incubating medium as detected by ELISA, was significantly reduced as compared to the controls treated with PBS (Figure 2A). Moreover, activation of ENaC by trypsin (20 μg/ml, 15 mins) induced abundant increases in PGE_2_ release into the medium, which was also significantly blocked by MK-571 (10 μM), suggesting a role of MRP4 in mediating PGE_2_ release from endometrial epithelial cells upon ENaC activation at embryo implantation (Figure 2A). It should be noted that epithelial PGE_2_ release into the stroma is a particularly important signal leading to stromal decidualization required for embryo implantation. Using immunofluorescence labeling, we found that MRP4 expression was specifically localized to the basolateral membrane of the endometrial epithelial cells, the interface between the epithelium and the stroma (Figure 2B). The polarized expression pattern of MRP4 and its capacity in PGE_2_ transport suggested an important role of MRP4 in transporting PGE_2_ from epithelial cells to the stromal cells to induce decidualization required for embryo implantation.

### Involvement of MRP4 in ENaC-activated CREB/COX-2 signaling and embryo implantation gene expression

**Figure 3:**
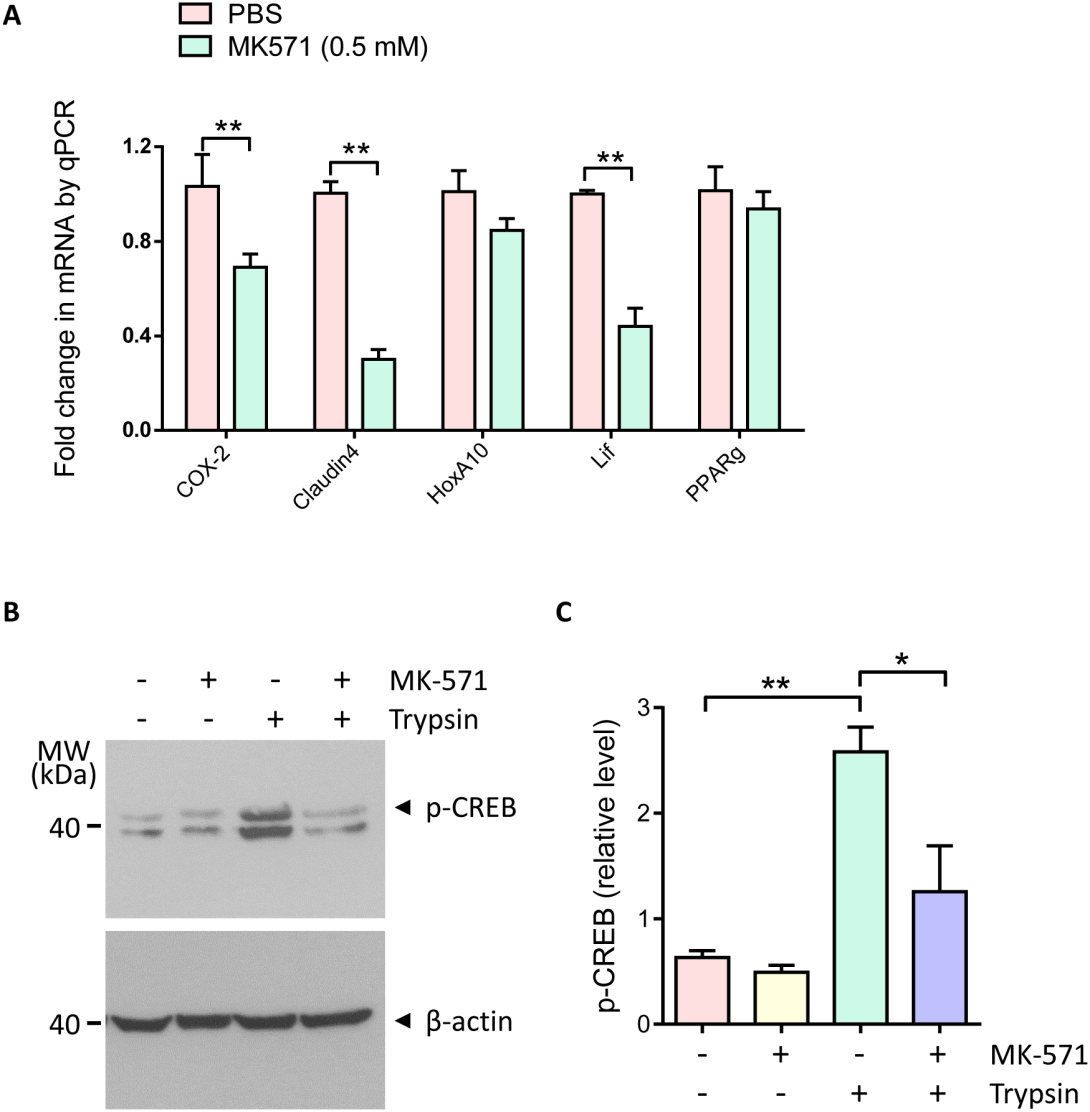
Involvement of MRP4 in regulation of embryo implantation gene expression and CREB/COX-2 signaling **(A)** Quantitative PCR of COX-2, Claudin4, HoxA10, Lif and PPARg in mouse uteri 24 hours after intrauterine injection with MK-571 (0.5 mM, day 3 post mating) (n = 4, ** P < 0.01). **(B-C)** Western blots **(B)** with quantification **(C)** for phosphorylated CREB (p-CREB) in human endometrial epithelial cells after incubated with MK-571 (10 μM) in the absence or presence of trypsin (20 μg/ml) (n = 3, * P < 0.05, ** P < 0.01).

Embryo implantation is a complicated process involving a series of critical marker genes, including COX-2, Claudin4 [22], HoxA10 [23], Lif [24] and PPARg [25]. We explored the involvement of MRP4 in embryo implantation by examing the effect of its inhibitor MK-571 on uterine expression of these implantation related genes. To inhibit MRP4, MK-571 was intrauterinally injected in pregnant mice on day 3 (prior to implantation). Uterine expression of genes was examined 24 hour later on day 4. The results showed that COX-2, Claudin4 and Lif were significantly reduced when MRP4 was inhibited by intrauterine injection with MK-571 (0.5 mM) as compared to the control group Figure 3A). Since COX-2 is a key enzyme in PGE_2_ synthesis and its transcription is known to be activated by ENaC-dependent phosphorylation of CREB during embryo implantation [10, 26], we next examined whether MRP4 may be involved in CREB phosphorylation by ENaC activation in endometrial epithelial cells. As shown in Figure 3B&3C, the level of phosphorylated CREB was significantly increased after treatment with trypsin, which was abolished by the treatment with MK-571 (10 μM) compared with that of the control group. Thus, these results suggest the involvement of MRP4 in ENaC-dependent CREB/COX-2 signaling during embryo implantation.

### Inhibition of MRP4 reduces implantation rate in mice

**Figure 4:**
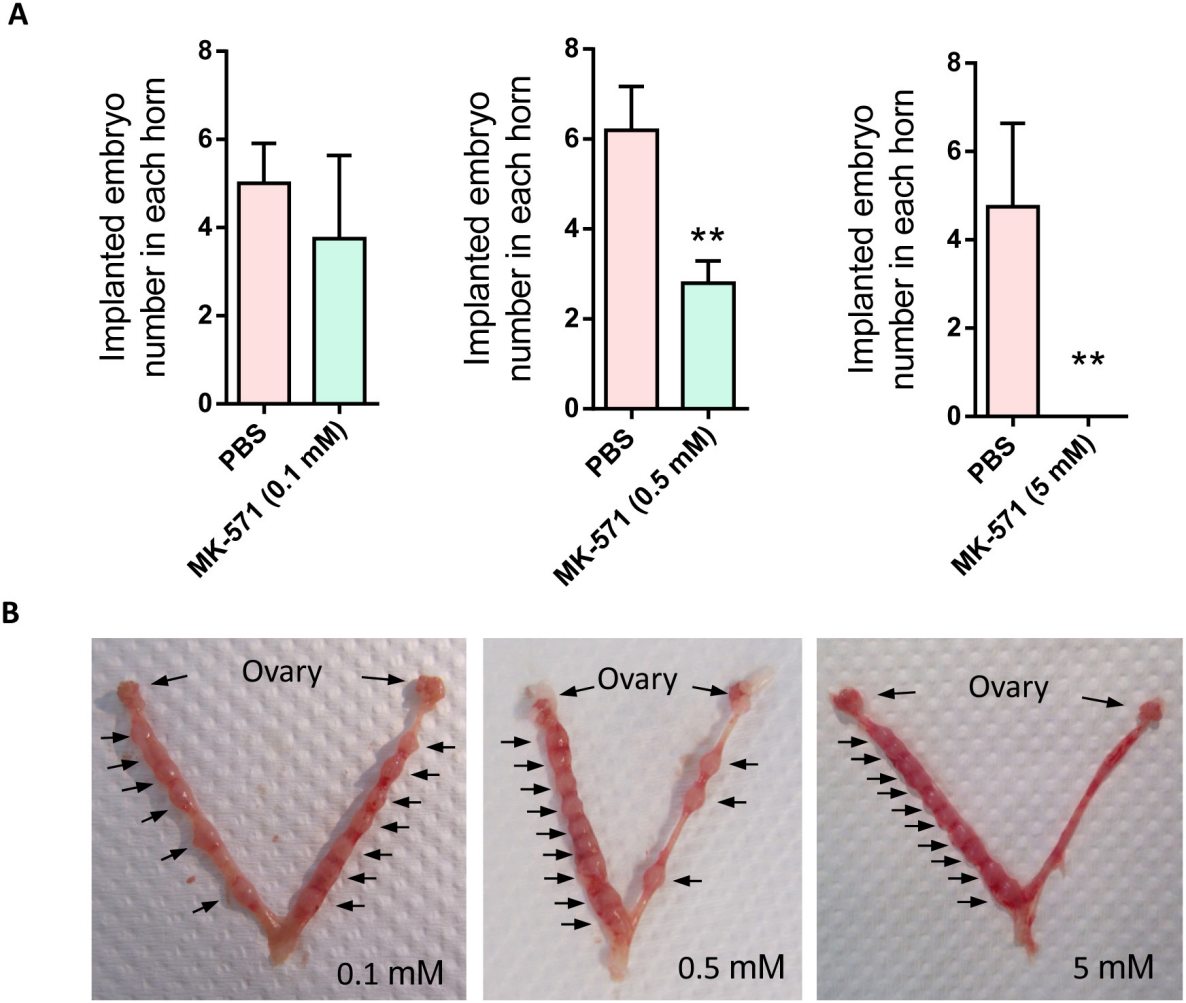
Effect of MRP4 inhibition on implantation rate in mice **(A-B)** Effect of intrauterine injection of MK-571 (0.1-5 mM, day 3) on implantation rate in mice with representative photographs **(B)** showing implantation sites (arrows) in the control uterine horns (left) and the MK-571 treated ones (right) (n = 3 - 5, ** P < 0.01 compared to Ctrl).

To confirm that PGE_2_ transport function of MRP4 indeed plays a role in embryo implantation, we counted the implantation rate on day 7, when the implanted sites are visible, after intrauterine injection of MK-571 on day 3 in the mouse *in vivo* model. The results showed that MK-571 (0.1 - 5 mM) dose dependently decreased the number of implanted embryos in the uterine horns compared to the vehicle treated control horns, as counted on day 7 (Figure 4A&4B). The results therefore suggested essential involvement of MRP4 in embryo implantation.

## DISCUSSION

The present study has identified a previously unexplored physiological role of MRP4 in mediating ENaC activation-triggered PGE_2_ production and release required for embryo implantation.

Despite the well-recognized role of PGE_2_ in embryo implantation, the study on the transport of PGE_2_ from uterine epithelium to stroma during embryo implantation is scarce. The present study has provided evidence suggesting that MRP4 is an essential mediator for the efflux or release of PGE_2_ from endometrial epithelial cells to the stroma required for embryo implantation. First, the expression of MRP4 in endometrial epithelial cells is upregulated during implantation and by ENaC activation induced by embryo-derived proteases, suggesting its responsiveness to embryo-derived signal. Second, MRP4 mediates epithelial PGE_2_ release triggered by ENaC activation. Importantly, the expression of MRP4 in the endometrial epithelial cells is polarized and predominately localized to the basolateral membrane, indicating the direction of PGE_2_ efflux toward the stroma beneath the epithelium. Interestingly, another known PG transporter (PGT), which only conducts the influx of PGE_2_ [27], has been observed to be strongly expressed in subluminal stroma cells at implantation sites in mice [28], suggesting an uptake of PGE_2_ in stroma cells at implantation. The differential expression pattern of these PGE_2_ transporters is consistent with a signal (PGE_2_) transfer from the epithelium to the stroma essential required for embryo implantation.

The present results also suggest the involvement of MRP4 in ENaC-dependent CREB phosphorylation. As we previously demonstrated, phosphorylated CREB may contribute to enhancement of PGE_2_ production in endometrial epithelial cells in two folds. First, it directly triggers the transcription of COX-2, a major enzyme for PGE_2_ synthesis in cells [10]. Second, it represses the expression of two COX-2 inhibiting microRNAs miR101 and miR199a and indirectly promote COX-2 expression [11]. More, other studies have suggested that CREB also represses 15-prostaglandin dehydrogenase (15-PDGH), which catalyzes the inactivation of PGE_2_ [29–31]. Therefore, the presently observed MRP4-dependent CREB phosphorylation may be another important mechanism regulating PGE_2_ production in the endometrial epithelial cells during embryo implantation. Of note, in addition to COX-2/PGE_2_, CREB seems to interact with other implantation genes such as Lif [32] and PPARg [33] in other systems, suggesting further role(s) of MRP4-regulated CREB in embryo implantation. A question remained how MRP4 contributes to CREB phosphorylation upon ENaC activation. We previously showed that the CREB phosphorylation is largely promoted by Ca^2+^ increase triggered by ENaC activation and depolarization [10]. Of note, CREB is responsive to both Ca^2+^ and cAMP. It has been reported that CREB phosphorylation can be evoked by PGE_2_ receptor E2 (EP2) activation and its coupled PKA signaling [34, 35]. Therefore, it is possible that MRP4-mediated PGE_2_ release may in turn promote CREB phosphorylation in epithelial cells. In this regard, MRP4, may not only mediate PGE_2_ release, but also contribute to a positive feedback that further enhances PGE_2_ production. However, the detailed underlying mechanism awaits further investigation.

The upregulation of MRP4 during embryo implantation, its responsiveness to ENaC activation by embryo-derived signal as well as its capacity in mediating PGE_2_ transport and promoting PGE_2_ production suggest an essential role of MRP4 in embryo implantation. This is confirmed by the present data obtained in the *in vivo* embryo implantation model showing that blockage of MRP4 function results in downregulation of implantation genes and implantation rate in mice. The present finding is consistent with the previous report showing a smaller little size in MRP4 knockout mice [20]. Of interest, the expression of MRP4 was reported in human eutopic and ectopic endometrial tissue and elevated in peritoneal endometriosis, which could be attenuated by the anti-inflammatory lipid lipoxin A(4) [36]. On the other hand, lipoxin A(4) was observed to block embryo implantation in mice [37]. In light of the present finding, the effect of lipoxinA(4) on embryo implantation could be due to its effect on downregulation of MRP4.

Like other members of MRP subfamily, MRP4 confers resistance to a wide variety of chemotherapeutic drugs [38, 39]. High levels of this gene are strongly predictive of poor outcome in cancers, including neuroblastoma, colorectal cancer and esophageal squamous cell carcinoma [18, 40, 41]. In addition, MRP4 has been shown to have remarkable ability to modulate cellular signaling processes besides its versatile efflux transport function as a potential therapeutic target in cancer treatment [17, 42]. It should be noted that there are striking similarities between the behavior of invasive blastocyst and that of cancer cells, such as migration, invasion and angiogenesis [43]. Indeed, the present study demonstrates a role of MRP4 in regulating ENaC-dependent CREB/COX-2/PGE_2_ signaling in embryo implantation. Similar finding has also been reported in esophageal squamous cell carcinoma [38]. Knockdown of MRP4 could attenuate the expression of p-CREB and COX-2, affecting PGE_2_ synthesis [41]; however, whether it involves ENaC was not investigated. Of note, the potential role of ENaC in the development and progression of multiple cancers has recently been recognized [44–47]. Therefore, the capacity of MRP4 in regulating ENaC-dependent signaling pathways during embryo implantation as demonstrated presently may provide new insights into the understanding of MRP4/ENaC related signaling in cancers as well. Given the multiple roles of both ENaC and CREB/COX-2/PGE_2_ signaling pathway in other physiological or pathological events, the role of MRP4 in regulation of ENaC-dependent CREB/COX-2/PGE_2_ signaling pathway may have far-reaching beyond embryo implantation.

## MATERIALS AND METHODS

### Mice and intrauterine injection

ICR mice were obtained from the Laboratory Animal Service Centre of the Chinese University of Hong Kong. All animal experiments were conducted in accordance with the university guidelines on animal experimentation, and approval by the Animal Ethics Committee of the Chinese University of Hong Kong was obtained for all related procedures. The day a vaginal plug was found after mating was identified as day 1. The intrauterine injection surgery under general anethesia was performed on day 3 after mating as previously reported [10]. MK-571 or vehicle control was injected into the lumen of each uterine horn close to the uterine-oviduct junction toward the uterine lumen. On day 7 after mating, the mice were killed by CO_2_ asphyxiation and implanted embryo numbers were counted. Uteri were collected for further analyses.

### Cell culture

The human endometrial epithelial cell line was provided as a gift from Dr. Douglas A. Kniss from the Laboratory of Perinatal Research at the Ohio State University. The cells were cultured in DMEM supplemented with 10% fetal bovine serum (v/v) and 1% penicillin-streptomycin (v/v) in 5% CO_2_ incubators at 37°C.

### RNA extraction and real-time PCR

Total RNA of cells were extracted using TRIzol reagent (Invitrogen Life Technologies) according to manufacturer’s instructions. 1 μg total RNA was applied on reverse transcription reaction using M-MLV reverse transcriptase (Promega) according to the manufacturer’s instructions. SYBR Green Master Mix (Tli RNase H Plus, Takara) was added to each PCR reaction along with cDNA and primers in a total volume of 10 μl. The primer sequences are listed in Table 1. Quantitative PCR were carried out in triplicate on a 96-well plate using an 7500 Fast Real-time PCR system (Applied Biosystems). The transcriptional expression of target genes were indicated with average CT value of GAPDH and calculated using the ΔΔC_T_ method.

**Table 1:** Primers used for RT-PCR

Gene	Primer	Sequence	Product size
Mouse	F	GGCGCAGTTTATGTTGTCTG	130 bp
COX-2	R	CAGCACTTCACCCATCAGTT	
Mouse	F	CCCTCATCAGTCACTCAGCA	140 bp
Claudin4	R	AGCAAACGTCCACTGTCCTT	
Mouse	F	GTGTAAGGGCAGCGTTTCTT	121 bp
HoxA10	R	CAGCCCCTTCAGAAAACAGT	
Mouse	F	AGCAGCAGTAAGGGCACAAT	117 bp
Lif	R	CCCCATTTGAGCATGAACTT	
Mouse	F	TGTCGGTTTCAGAAGTGCCTTG	121 bp
PPARg	R	TTCAGCTGGTCGATATCACTGGAG	

### Immunoblotting

The cells was lysed in ice-cold RIPA lysis buffer (50 mM Tris-Cl, pH 7.5, 150 mM NaCl, 1% NP-40, 0.5% DOC, 0.1% SDS) with protease and phosphatase inhibitor cocktail (catalog #78443, Thermo Scientific) for 30 min on ice. Supernatant was colleceted after centrifugation at 14,000 rpm for 30 min at 4°C. Equal amounts of protein were resolved by SDS-polyacrylamide gel electrophoresis and electroblotted onto equilibrated nitrocellulose membrane. After blocking with 5% milk, the membranes were immunodetected for target proteins. Antibodies against MRP4 (1:100, Abcam, ab15602); phospho-CREB (1:1000, Cell Signaling, 9198), β-tubulin (1:2000, Santa Cruz, sc-9104) and β-actin (1:5000, Sigma, A1978). The protein bands were detected with HRP-conjugated antibodies and visualized by the enhanced chemiluminescence (ECL) assay (GE Healthcare) following manufacturer’s instructions. Signals were quantified by Imagine J software and defined as the ratio of target protein relative to internal loading control.

### PGE_2_ ELISA

The human endometrial epithelial cells were grown in 24-well plates for 24 h. 1 % FBS in DMEM culture medium was used for 8 h to synchronize the cells before the experiment. After cell starvation, FBS-free DMEM medium was used for all the treatments and cell-free supernatant with PGE_2_ content was collected and measured using an EIA kit (Cayman Chemical, 514010).

### Tissue fixation and cryosectioning

Uterus tissue from day 4 of pregnancy mice after intrauterine injection were harvested and fixed by immersion in 4% paraformaldehyde overnight. After three times washed in PBS, uterus were cryoprotected in 30% sucrose in PBS at 4°C for 24 hours, mounted in OCT embedding media (Tissue-Tek, 4583, Sakura) and frozen at -80°C. Cryosection were cut to a thickness of 5 μm using a cryostats (Shandon Cryotome, Thermo Scientific) and placed onto Superfrost/Plus microscope slides (Fisher Scientific).

### Immunofluorescence

Sections were rehydrated in PBS for 5 mins, boiled in a microwave oven with citrate buffer (pH 6.0) for 20 mins and cooled down to room temperature and treated with 1% SDS in PBS for 4 mins. Sections were blocked with 1% bovine serum albumin (BSA) in PBS for 15 mins, incubated with primary antibody (MRP4, 1:20, Abcam, ab15602) overnight at 4°C and followed by incubation with fluorochrome-conjugated secondary antibody (invitrogen) for 1 h at room temperature. DAPI was used to stain cell nuclei. Images were acquired with a confocal microscope (Zeiss, Germany).

### Statistical analysis

The results are shown as mean ± SEM. Differences in measured variables between two-group comparison were assessed by using Student’s unpaired t-test. One-way ANOVA for multiple comparisons was applied for comparing more than two groups. A Value of P < 0.05 was considered to be statistically significant.
